# Crossmodal semantic congruence can affect visuo-spatial processing and activity of the fronto-parietal attention networks

**DOI:** 10.3389/fnint.2015.00045

**Published:** 2015-07-10

**Authors:** Serena Mastroberardino, Valerio Santangelo, Emiliano Macaluso

**Affiliations:** ^1^Neuroimaging Laboratory, Santa Lucia FoundationRome, Italy; ^2^Department of Philosophy, Social Sciences & Education, University of PerugiaPerugia, Italy

**Keywords:** attention, multisensory, semantics, space, fronto-parietal networks

## Abstract

Previous studies have shown that multisensory stimuli can contribute to attention control. Here we investigate whether irrelevant audio–visual stimuli can affect the processing of subsequent visual targets, in the absence of any direct bottom–up signals generated by low-level sensory changes and any goal-related associations between the multisensory stimuli and the visual targets. Each trial included two pictures (cat/dog), one in each visual hemifield, and a central sound that was semantically congruent with one of the two pictures (i.e., either “meow” or “woof” sound). These irrelevant audio–visual stimuli were followed by a visual target that appeared either where the congruent or the incongruent picture had been presented (valid/invalid trials). The visual target was a Gabor patch requiring an orientation discrimination judgment, allowing us to uncouple the visual task from the audio–visual stimuli. Behaviourally we found lower performance for invalid than valid trials, but only when the task demands were high (Gabor target presented together with a Gabor distractor vs. Gabor target alone). The fMRI analyses revealed greater activity for invalid than for valid trials in the dorsal and the ventral fronto-parietal attention networks. The dorsal network was recruited irrespective of task demands, while the ventral network was recruited only when task demands were high and target discrimination required additional top–down control. We propose that crossmodal semantic congruence generates a processing bias associated with the location of congruent picture, and that the presentation of the visual target on the opposite side required updating these processing priorities. We relate the activation of the attention networks to these updating operations. We conclude that the fronto-parietal networks mediate the influence of crossmodal semantic congruence on visuo-spatial processing, even in the absence of any low-level sensory cue and any goal-driven task associations.

## Introduction

Over the last 30 years, multisensory processing and the integration of signals across sensory modalities has gained much interest (for a review see Calvert, 2001; see also Stevenson et al., 2014). An outstanding issue in this field concerns to what extent crossmodal interactions occur in a fully automatic manner or whether there are significant couplings between multisensory processing and attention control. While traditional views emphasized pre-attentive mechanisms of multisensory integration, recent studies highlighted that attention and multisensory processing can influence each other in many different ways (McDonald et al., 2001; Koelewijn et al., 2010; Talsma et al., 2010; Santangelo and Macaluso, 2012, for reviews). Here we sought to contribute to this debate by asking whether crossmodal semantic congruence between visual and auditory signals presented at different locations can generate spatial attention biases and affect the processing of subsequent visual stimuli. Specifically, we made use of a paradigm where the audio–visual signals were fully task-irrelevant and did not provide any low-level spatial cues that might affect the processing of the visual targets. Therefore, any crossmodal spatial influence on visual processing can be attributed to crossmodal semantic processing rather than other low-level/bottom–up or goal-related factors directly linking the multisensory input to visual-spatial attention control processes.

Previous studies have shown that auditory stimuli can affect visual spatial processing, consistent with supramodal mechanisms of attention control (e.g., Vecera and Farah, 1994). Crossmodal spatial cueing studies have shown that a lateralised auditory stimulus (non-predictive cue) can influence the response to a subsequent visual target, with better performance when the target is presented at the same location as the cue (valid trials) than on the opposite side (invalid trials; Driver, 1996; Spence and Driver, 1997, 1998; McDonald et al., 2000). These crossmodal cueing effects suggest that a sudden auditory onset at one location can attract visual attention toward that location, and that processing targets on the opposite side requires additional processes (e.g., disengaging from the cued location, shifting/re-orienting, and re-engaging at the position of the visual target, see Posner and Cohen, 1984; Brown and Denney, 2007; Chen, 2012). Other studies have highlighted the influence of spatially non-informative auditory cues on visual search tasks. One example of this is the “pip-and-pop” effect, where the binaural presentation of a sound synchronized with a color change of the visual target can boost search performance (Van der Burg et al., 2008; van den Brink et al., 2014). While cueing and search paradigms differ in many ways (e.g., role of temporal vs. spatial correspondences between the two modalities), they both rely on spatially localized low-level changes in the sensory input. Indeed, one possible mechanism generating these crossmodal interactions is that the between-modalities (spatial and/or temporal) correspondence of the physical change makes the target location more salient via bottom–up, stimulus-driven attention control (e.g., Van der Burg et al., 2008; see also Talsma et al., 2010, for review).

However, high-level factors can also contribute to crossmodal influences on visuo-spatial processing. For example, semantic congruence plays an important role during the processing of complex audio–visual stimuli and has been found to influence visual attention. Using a search task, Iordanescu et al. (2008) presented pictures of natural objects/animals together with a centrally presented non-informative sound. They found faster target localization when the target object (e.g., a picture of a cat) was presented together with a semantically congruent sound (i.e., a meow), compared with an unrelated sound or a sound associated with a distractor picture (see also Iordanescu et al., 2010). These findings suggest that audio–visual semantic congruence can bias visuo-spatial processing, e.g., via enhanced representation of the visual target (Iordanescu et al., 2008, 2010), even in the absence of any spatially localized sensory change linking the central sound and the visual target. However, in these visual search studies and the pip-and-pop effect studies, the visual component of the “interacting” audio–visual stimuli was always task-relevant (i.e., a visual target). An exception to this is Experiment 5 in Van der Burg et al. (2008) that revealed a marginal effect/cost for sounds coupled with distractors. However, it should be considered that during serial search, participants will voluntarily shift attention between the various elements of the visual display, including the distractors. Therefore, audio–visual interactions for sounds synchronized with a distractor-change will sometimes involve visual stimuli (i.e., the synchronized distractor) that might be attended to in a goal-driven manner.

In the studies discussed above, goal-driven attention was directed toward the multisensory stimuli (or – at least – the visual component of these), which is likely to have a significant impact on how/whether the two modalities interacted with each other (see below; and Koelewijn et al., 2010, for a review). In the context of cueing studies, one approach to assess whether crossmodal spatial interactions also occur between task-irrelevant stimuli consists in using bimodal non-predictive cues. For example, Santangelo et al. (2006) presented audio–visual cues followed by unimodal visual targets. They found that spatially congruent bimodal cues on the same side of the visual target lead to faster discriminations, but this effect was not larger than the cueing effect elicited by unisensory auditory or visual cues. While this null finding suggests that audio–visual stimuli do not interact with each other when fully task-irrelevant, later studies showed that bimodal cues can affect ERPs over and above any effect of unimodal cues (Santangelo et al., 2008b) and that, unlike unimodal cues, they influence visual target discrimination also under high-load, dual-task conditions (Santangelo and Spence, 2007; Santangelo et al., 2008a; see, for a review, Santangelo and Spence, 2008). Additional evidence for the influence of irrelevant audio–visual stimuli on visuo-spatial processing comes from a study by Matusz and Eimer (2011). In this study, each trial included a first array of irrelevant visual stimuli coupled with a centrally presented sound, followed by a visual search display. The results showed improved search performance when the sound was coupled with a color change in the first display, at the same location of the subsequent visual target. This effect did not depend on the relationship between the color of the cue and the currently relevant target color (cf. contingent attentional capture, Folk et al., 1992), consistent with pure bottom–up mechanisms of attentional capture. In a subsequent study, Matusz et al. (2015) further investigated the possible influence of top–down signals during the processing of irrelevant audio–visual stimuli, now using semantic matching. Each trial required the discrimination of a visual target that was either presented in isolation (low top–down task demand) or embedded in visual distractors (high demand). In the critical audio–visual distractor trials, a task-irrelevant colored visual stimulus was presented together with a voice saying the color (e.g., a red square shape coupled with a spoken “red”). In adult participants, the results showed that task-irrelevant audio–visual stimuli that included goal-relevant information (e.g., the color “red” was also a relevant feature of the target) interfered with target discrimination irrespective of task demands. In summary, several studies have demonstrated the influence of irrelevant audio–visual stimuli on visual attention, but they have always involved either spatially localized “bottom–up” physical changes in the sensory input (e.g., Santangelo and Spence, 2007; Santangelo et al., 2008a; Matusz and Eimer, 2011); or shared features between the audio–visual stimuli and the task-relevant visual target (interfering distractors, in Matusz et al., 2015).

Accordingly, the main aim of the current study was to investigate crossmodal spatial influences of task-irrelevant audio–visual stimuli on visual processing, in the absence of any low-level spatial cue related to the onset of the stimuli, or any goal-related signal linking the audio–visual stimuli with the subject’s current task. For this, we presented sounds together with semantically related/unrelated pictures (cf., Iordanescu et al., 2008), which were completely irrelevant to participants’ task. The task of the participants was to perform an orientation discrimination of a Gabor patch presented after the audio–visual stimuli. The target Gabor patch was presented either on the same side (valid trails) or on the opposite side (invalid trials) of the picture that was semantically congruent with the centrally presented sound. We hypothesized that the semantic relationship between the central sound and one picture would influence visuo-spatial attention, which in turn would affect the processing of the subsequent visual targets.

From the neuroimaging perspective, several previous studies have investigated the neural substrate of crossmodal semantic congruence by presenting in-/congruent pictures and sounds (e.g., Taylor et al., 2006; Noppeney et al., 2007; see Doehrmann and Naumer, 2008, for a review). These studies highlighted that audio–visual semantic interactions can affect activity in polysensory regions of the superior temporal sulcus, as well as higher-order areas in the medial temporal cortex and the left prefrontal cortex. In the current study, we might expect the involvement of these brain areas, but note that our main “valid/invalid” comparisons entailed trials with identical audio–visual input, with one picture that is always congruent with the sound and one that is incongruent. Because of this, areas involved in audio–visual semantic matching are unlikely to show any differential condition-specific effect. In contrast, because we expected crossmodal interactions to influence visuo-spatial processing, we would predict condition-specific effects in fronto-parietal networks associated with visuo-spatial attention control (Corbetta and Shulman, 2002). These networks have also been found to activate in studies of attention control in modalities other than vision (e.g., see Yantis et al., 2002; Macaluso et al., 2003; Krumbholz et al., 2009; Hill and Miller, 2010, for the dorsal network; and Downar et al., 2000; Macaluso et al., 2002, for the ventral network), which makes them the ideal candidates for mediating the influence of non-visual signals on visuo-spatial attention control.

In the current paradigm, we aimed to uncouple the audio–visual stimuli from any goal-related signals associated with the subject’s task, and we eliminated any stimulus-driven spatial cue by avoiding spatially localized sensory changes when presenting the audio–visual stimuli (see above). Arguably, the semantic matching of the sound with the semantically related picture still entails endogenous processes such as internal object representations required to combine visual and auditory signals (Iordanescu et al., 2008; see also Fiebelkorn et al., 2010). These endogenous effects should be distinguished from more traditional goal-directed processes associated, for example, with predictive cues that provide participants with task-relevant information (i.e., signaling the most likely location of the up-coming target) and can be used to strategically control spatial attention in a goal-driven manner. Nonetheless, the involvement of endogenous processes for crossmodal semantic matching and our main expectation that these will affect processing of the task-relevant visual targets lead us to hypothesize the involvement of dorsal fronto-parietal regions associated with top–down control (Corbetta and Shulman, 2002), as well as ventral regions where top–down and stimulus-driven signals jointly contribute to visuo-spatial orienting (Corbetta et al., 2008; Geng and Vossel, 2013; Macaluso and Doricchi, 2013; see also Natale et al., 2009, for a fMRI study using non-predictive cues but involving top–down control).

**FIGURE 1:**
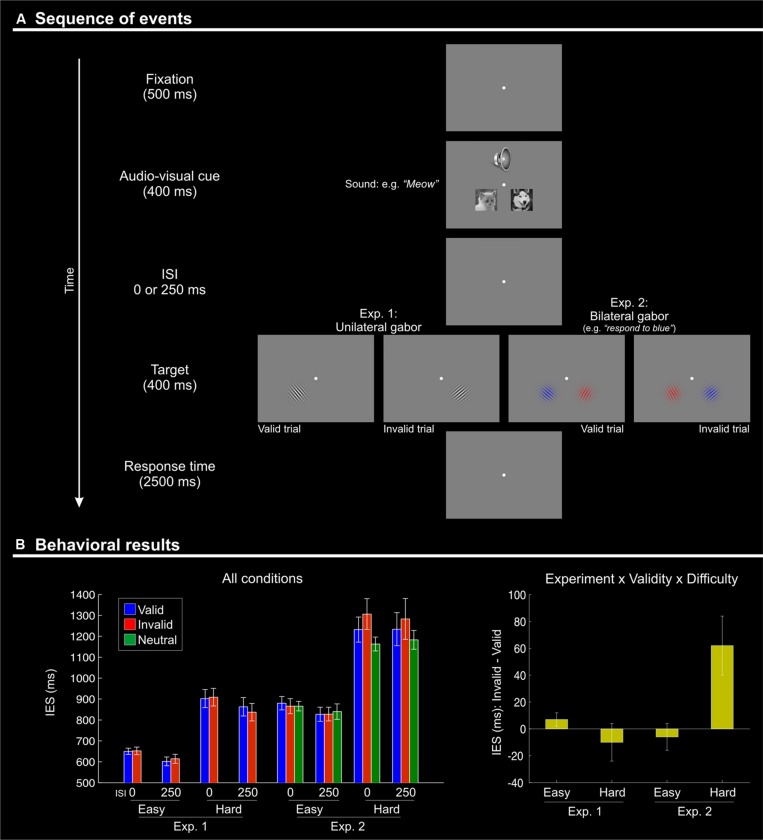
**(A)** Schematic diagram showing the sequence of events during one trial. Each trial began with the presentation of a fixation point for 500 ms. Two pictures (cat and dog) were then presented simultaneously with a centrally presented sound: a “meow” or a “woof”. The stimuli in both modalities lasted for 400 ms. After a variable inter-stimulus interval (ISI) of 0 or 250 ms, a single (Experiment 1) or two (one in each visual hemifield; Experiment 2) Gabor patches were presented for 400 ms. Subjects had 2500 ms to respond to the orientation of the single Gabor in Experiment 1, or to the target Gabor of the relevant color in Experiment 2. Visual targets appeared either in the location of the picture that was semantically congruent with the sound (“valid”) or on the other side (“invalid”). After an inter-trial interval (ITI) of 1500 ms with a blank screen, a new trial began. **(B)** Behavioral results (inverse efficiency scores, IES) showing a Validity effect (invalid > valid) only for the difficult orientation discrimination trials (“hard” conditions) of Experiment 2. Left panel: mean IES plotted separately for each experimental condition; Right panel: the Validity effect (invalid – valid) plotted separately for “easy” and “hard” conditions, in the two experiments. The selective effect of Validity in the “hard” condition of Experiment 2 (cf. rightmost bar in this plot) highlights the 3-way interaction between Validity, Difficulty, and Experiment.

In order to gain further insights into the relative contributions of top–down and stimulus-driven control in the current paradigm, the design included several additional manipulations. First, we varied the time between the offset of the irrelevant audio–visual stimuli and the onset of the visual target (ISIs = 0 or 250 ms). We expected that if audio–visual semantic matching generates spatial signals analogous to those typically associated with non-predictive peripheral cues, maximal effects should occur with the shortest ISI. In contrast, if semantic matching generates a top–down signal analogous to goal-related signals typically associated with predictive cues, the effects should be largest with the longer ISI (e.g., Ruz and Lupiáñez, 2002; and Rauschenberger, 2003, for reviews). Second, we manipulated top–down task demands by varying the general difficulty of target discrimination (i.e., easy vs. difficult tilt judgment, see **Figure 1A**) and by varying the amount of spatial competition during the visual judgment task (target Gabor only, in Experiment 1; target plus one distractor Gabor in Experiment 2; see **Figure 1A**). When two Gabor patches were presented, the participants had to make use of internal information about the current task-set (i.e., a relevant color defining the target Gabor), which implies additional top–down control during the target phase of the trial (see Indovina and Macaluso, 2007, who used an analogous procedure to demonstrate the role of top–down control for the activation of the inferior parietal cortex in a purely visual task). Based on previous studies that used dual-task procedures to engage processing resources away from multisensory stimuli (e.g., Alsius et al., 2005; Santangelo and Spence, 2007; and Koelewijn et al., 2010, for a review), here one might predict a reduction of any crossmodal effect of semantic congruence in conditions of high task-demands (see also Eimer and Kiss, 2008; for a purely visual study showing that changes of target-related task demands can influence effects associated with preceding non-predictive cues, albeit in the context of a contingent capture paradigm). However, if the multisensory stimuli impact top–down control mechanisms engaged only under high task demands, one might expect crossmodal influences specifically in conditions engaging these additional control processes.

In summary, we asked whether audio–visual semantic congruence can bias the processing of subsequent visual targets, specifically when the audio–visual stimuli are task-irrelevant and do not generate any spatially localized sensory change. We presented two pictures, one in the left and one in the right hemifield, together with a central sound that was semantically congruent with one of the two pictures. Shortly after, we presented the visual target either on the side of the picture congruent with the central sound (valid trials) or on the opposite side (invalid trials). We hypothesized that the picture-sound semantic congruence would affect visuo-spatial attention and, thus, that the processing of the subsequent visual target would change as a function of the trial in-/validity. We predicted that these crossmodal effects would impact primarily on the activity of the fronto-parietal attention networks, where any spatial signal associated with semantic congruence may interact with other task-related factors that regulate the functioning of these attention control systems.

## Materials and Methods

### Participants

Nineteen volunteers participated in Experiment 1 and 20 in Experiment 2. None of the subjects participated in both experiments. All participants were neurologically intact, were not on psychotropic or vasoactive medication, and had no history of psychiatric or neurological disease. They had normal or corrected-to-normal vision (i.e., with contact lenses) and reported normal hearing. Before scanning, all participants were tested in a 20-min training session. The training session comprised one block of 192 trials. The stimuli and the task were identical to those presented during the imaging sessions (see Stimuli and Procedure), with the exception that at the end of each trial the participant received visual feedback of his/her performance. Subjects who failed to reach 80% accuracy in the training session did not participate in the imaging experiment (*n* = 2 in Experiment 1, and *n* = 4 in Experiment 2). Four subjects were excluded from data analysis of Experiment 2 for excessive within-fMRI-run head movements (larger than 2 mm or 2°). Therefore, the final analyses included 17 participants for Experiment 1 (7 males; mean age = 22.3 ± 3.3) and 12 participants for Experiment 2 (7 males; mean age = 24.3 ± 3.0). All subjects gave written informed consent to participate in the study, which was approved by the independent Ethics Committee of the Santa Lucia Foundation (Scientific Institute for Research Hospitalization and Health Care).

### Stimuli and Procedure

Stimulus presentation was controlled with Matlab 7.1 (The MathWorks Inc., Natick, MA, USA), using the Cogent2000 Toolbox (Wellcome laboratory of Neurobiology, University College London). Visual stimuli were presented on a gray background using a rear projection system. Participants were instructed to maintain fixation on a central dot during the scanning sessions. The auditory stimuli were presented using MRI compatible headphones.

In both experiments, the participants were presented with two pictures (cat or dog), one on each side of the central fixation point, plus an auditory stimulus presented binaurally. The pictures and the sound were presented for 400 ms, which should be sufficient time to identify the audio–visual object before the onset of the subsequent visual target (e.g., see De Lucia et al., 2010, who showed neuro-physiological signatures of auditory categorization of complex sounds at around 200 ms post-stimulus onset, even with a large pool of 160 stimuli rather than just 2–3 sounds used here). The pictures were displayed in black and white (resolution 200 × 200 pixels), centerd 2.7° to the left and to right of the central fixation and 2° below it, subtending a visual angle of 3.8° × 3.8° (see **Figure 1A**). The auditory stimulus consisted of a cat’s meow or a dog’s bark, presented binaurally and perceived centrally. Experiment 2 included a third sound type (a frog’s croak) that was used for the “neutral” trials (see below).

After a variable delay (ISI = 0 or 250 ms), the participant was presented with the visual target. In Experiment 1, the target was a single Gabor patch (visual angle size 3.8° × 3.8°) presented in either the left or right hemifield, in place of the picture of the cat or the dog. In the *valid* conditions, the target was presented on the side where the picture was congruent with the sound (e.g., Gabor on the left when preceded by a picture of a cat on the left coupled with a “meow” sound). In the *invalid* conditions, the target was presented on the opposite side of the congruent picture (e.g., Gabor on the left when preceded by a picture of a dog on the right and a “woof” sound). The position of the congruent picture was not predictive of the target position (i.e., 50% “cue validity”). Despite this, participants might have sought to find a systematic relationship between the position of the congruent picture and the visual target, thus using the irrelevant audio–visual stimuli to direct spatial attention in a goal-driven manner. While this cannot be excluded, it should be emphasized that all participants underwent a 20-min training session, and it is unlikely that they continued looking for this inexistent relationship throughout the whole fMRI experiment.

On each trial, the target Gabor patch had one out of eight possible orientations. The task of the subject was to discriminate whether the Gabor orientation was smaller or larger than 45° and to report this by pressing a button either with the index finger (larger) or with the middle finger (smaller). In the *easy* conditions, the target was oriented at either 30 or ±60°; while in the *hard* conditions the tilt was either ±40° or ±50°. Subjects had 2500 ms to provide a response. This was followed by a variable inter-trial interval (1–3 s, uniformly distributed). Overall, Experiment 1 comprised 384 trials, equally divided in three fMRI runs. Each run of 128 trials comprised 16 repetitions of each of the eight experimental conditions [2 (Validity) × 2 (ISI) × 2 (Difficulty)].

In Experiment 2, the target phase of the trial comprised the presentation of two Gabor patches, flashed simultaneously in the left and right visual fields. One patch was colored in red while the other was in blue (see **Figure 1A**, panels on the right). Before starting the experiment, the participants were instructed which color of the Gabor patch was task relevant (counterbalanced across participants). This defined the location of the visual target that required discrimination and response and, thus, whether the trial was valid or invalid. Experiment 2 also included a *neutral* condition, where the cat/dog pictures were coupled with the sound of a frog’s croak. This neutral condition allowed us to address the additional question of whether any effect of Validity (invalid vs. valid trials) resulted from a cueing “benefit” on valid trails, a “cost” on invalid trials, or both. Valid, invalid and neutral cues were equally likely and not predictive of target location. Experiment 2 comprised a total of 576 trials, equally divided into three fMRI runs. In each run of 192 trials, each of the 12 experimental conditions [3 (Validity) × 2 (ISI) × 2 (Difficulty)] was repeated 16 times.

### Eye Movement Recording

To make sure that participants maintained central fixation through the experimental sessions, eye position was monitored using an infrared ASL eye-tracking system, adapted for use in the scanner (Applied Science Laboratories, Bedford, MA, USA; Model 504, sampling rate 60 Hz). Changes in horizontal eye position greater than ±2° of visual angle in a time window of 1550 ms (i.e., from trial onset to target offset, inclusive of the longer ISI of 250 ms) were classified as failure to maintain fixation. Overall, participants made few eye movements away from central fixation (4% Experiment 1; 5% Experiment 2).

### Magnetic Resonance Imaging

A Siemens Allegra (Siemens Medical Systems, Erlangen, Germany) operating at 3T and equipped for echo-planar imaging (EPI) was used to acquire the functional magnetic resonance images. A quadrature volume head coil was used for radio frequency transmission and reception. Head movement was minimized by mild restraint with cushions. Thirty-two slices of functional MR images were acquired using blood oxygenation level-dependent imaging (3 mm × 3 mm, 2.5 mm thick, 50% distance factor, repetition time = 2.08 s, time echo = 30 ms), covering the entirety of the cortex.

Image pre-processing and data analysis were performed using SPM8 (Wellcome Department of Cognitive Neurology) implemented in Matlab 7.1 (The MathWorks Inc., Natick, MA, USA). In Experiment 1, we collected a total of 885 fMRI volumes (295 × 3 runs); while in Experiment 2 we collected a total of 1275 fMRI volumes (425 × 3 runs). For each participant, after having discarded the first four volumes of each run, all images were corrected for head movements. All images were normalized using the SPM8 standard EPI template, re-sampled to a 2-mm isotropic voxel size and spatially smoothed using an isotropic Gaussian kernel of 8 mm FWHM. The time series at each voxel for each participant was high-pass filtered at 220 s and pre-whitened by means of the autoregressive model AR (1) (Friston et al., 2002).

For statistical inference, we used a random effects approach (Penny and Holmes, 2004). This comprised two steps. First, for each participant the time series at each voxel was best-fitted by model parameters based on a linear combination of effects of interest. These were delta functions representing: for Experiment 1, the onsets of the eight conditions given by our 2 × 2 × 2 factorial design [Validity (valid, invalid); Difficulty (easy, hard); ISI [0, 250)]; and for Experiment 2, the onsets of the 12 conditions of our 3 × 2 × 3 factorial design [Validity (valid, invalid, neutral); Difficulty (easy, hard); ISI (0, 250)]. All onsets were convolved with the SPM8 hemodynamic response function. The onset of the hemodynamic response function was aligned with the onset of the multisensory cue, with duration = 0. Onsets of trials in which an erroneous response occurred were included in the general linear model as covariates of no interest, and excluded from any further analysis of the imaging data.

For statistical inference at the group level, we considered the data of both experiments together, allowing us to formally assess the effect of Validity (invalid vs. valid trials) as a function of task-demands (high vs. low in Experiment 2 vs. Experiment 1, respectively)]. We tested for the main effect of Validity (invalid vs. valid) and any interaction between this and the factors associated with top–down control (i.e., ISI, Difficulty, and Experiment) in ANOVAs. For each participant, we computed the contrast “invalid minus valid trials” separately for the two ISIs (0, 250 ms) and the two levels of discrimination Difficulty (easy, hard). The resulting four conditions/effects per subject were entered in the AVOVA that included the Validity effects of both groups (Experiment 1 and 2) and enabled us to test our main hypothesis of the effect of crossmodal semantic congruence on visuo-spatial attention: i.e., the overall effect of Validity (invalid > valid) and any modulation by the three top–down factors (e.g., larger re-orienting effects when the task required focused spatial selection: (invalid – valid) _Exp2_ > (invalid – valid) _Exp1_; i.e., the interaction “Validity × Experiment”).

In addition, we carried out two separate ANOVAs that tested for the effects of ISIs and discrimination Difficulty, irrespective of Validity. For each participant, we computed the contrast ISI “0 - 250” (irrespective of Validity and Difficulty); and the contrast “hard minus easy” (irrespective of Validity and ISI). These were entered in two separate ANOVAs, where we tested for the mean effect of ISI/Difficulty across Experiments, and the interactions between ISI/Difficulty and Experiment.

Finally, for Experiment 2 only, we compared the overall effect of “valid and invalid cues” against the “neutral cues” in a separate group analysis. First, for each subject, we computed the contrast “[(valid + invalid)/2] minus neutral” separately for the two ISIs and the two levels of task Difficulty. We then ran another ANOVA to test for the overall effect of “valid/invalid versus neutral” trials and for any interaction between this and the other two factors included in Experiment 2 (i.e., ISI and task Difficulty).

All ANOVAs were corrected for non-sphericity (Friston et al., 2002) to account for possible differences in error variance across conditions. Statistical thresholds were set to *p*-FWE-corrected (family wise error) = 0.05 at cluster level (cluster size estimated at *p*-uncorrected, *p*-uncorrected = 0.005), considering the whole brain as the volume of interest.

## Results

### Behavioral Data

To allow comparisons among conditions accounting for any possible effect of speed-accuracy tradeoffs, we computed and analyzed inverse efficiency scores (IES; Townsend and Ashby, 1983; see also Bruyer and Brysbaert, 2011). For completeness, we also report the analyses of the reaction times and the error rates (RTs and ER, see legend of **Table 1**). The behavioral data were analyzed with SPSS (Statistical Package for Social Science, version 13.0). The Greenhouse-Geisser procedure was used to correct for any violations of sphericity. Analogous to the imaging analyses, the main behavioral analysis considered the two experiments together allowing us to test for condition-by-experiment interactions (see also Magnetic Resonance Imaging, above).

**Table 1:** Behavioral data.

		Difficulty: Easy						Difficulty: Hard					
		ISI 0			ISI 250			ISI 0			ISI 250		
		Valid	Invalid	Neutral	Valid	Invalid	Neutral	Valid	Invalid	Neutral	Valid	Invalid	Neutral
Experiment 1	IES(ms)	650 ± 15	652 ± 18		602 ± 21	614 ± 22		902 ± 43	909 ± 42		863 ± 44	837 ± 42	
	RT(ms)	633 ± 17	631 ± 17		584 ± 20	589 ± 20		715 ± 26	720 ± 29		67031 ±	671 ± 28	
	ER(%)	3 ± 1	3 ± 2		3 ± 1	4 ± 1		20 ± 2	20 ± 3		22 ± 2	19 ± 3	
Experiment 2	IES(ms)	880 ± 32	866 ± 36	866 ± 23	827 ± 34	828 ± 33	840 ± 37	1232 ± 60	1306 ± 74	1163 ± 33	1234 ± 79	1283 ± 98	1183 ± 45
	RT(ms)	840 ± 29	832 ± 31	838 ± 23	802 ± 36	799 ± 33	805 ± 31	973 ± 44	995 ± 52	929 ± 26	968 ± 57	961 ± 62	906 ± 24
	ER(%)	4 ± 1	4 ± 2	3 ± 1	3 ± 1	3 ± 1	4 ± 1	21 ± 2	23 ± 3	20 ± 3	21 ± 2	24 ± 3	22 ± 3

*Mean (±SEs) inverse efficiency scores (IES), reaction times (RTs), and error rates (proportion), for the different conditions in the two Experiments. RTs and ERs were tested in a four-way mixed ANOVA with the factors of: Validity (valid, invalid), Difficulty (easy vs. hard), ISI (0 vs. 250 ms), and Experiment (1 vs. 2). This revealed main effects of Experiment [RT means: 652 vs. 896 ms, *F*(1,27) = 32.1, *p* < 0.001; ER means: 12 vs. 13%, *F*(1,27) < 1, n.s.]; main effects of Difficulty [RT: 714 vs. 834 ms, *F*(1,27) = 60.5, *p* < 0.001; ER: 3 vs. 21%, *F*(1,27) = 227.7, *p* < 0.001]; a main effect of ISI, for RT only [RT: 793 vs. 755 ms, *F*(1,27) = 25.6, *p* < 0.001; ER: 12 vs. 12%, *F*(1,27) < 1, n.s.]; without any significant main effect of Validity. However, we found a three-way interaction between Validity, Experiment, and Difficulty for the ER [*F*(1,27) = 7.2, *p* = 0.013], but not for RT [*F*(1,27) < 1, n.s.], see also the main text for the results of the IES analyses. Val/Inv/Neu, valid/invalid/neutral trials.*

The IES are shown in **Figure 1B** and in **Table 1**. We carried out a four-way mixed ANOVA with “Experiment” as a between-subjects factor (1 vs. 2) and the following three within-subjects factors: “Validity” (valid, invalid), “Difficulty” (easy, hard), and “ISI” (0, 250 ms). The ANOVA revealed significant main effects of “Experiment,” “Difficulty,” and “ISI.” Participants were more accurate and faster in judging the target in Experiment 1 than in Experiment 2 {IES means: 754 vs. 1057 ms; [*F*(1,27) = 31.7, *p* < 0.001]}. Discrimination performance was better for easy compared to hard trials {IES: 740 vs. 1071 ms, [*F*(1,27) = 166.3, *p* < 0.001]}, and a decrease in discrimination performance was found for short compared to long ISIs (IES means: 925 vs. 866 ms; [*F*(1,27) = 7.3, *p* = 0.012]). Analogous results were obtained for the RT data (see **Table 1**).

While the main effect of “Validity” was not significant, the ANOVA revealed a significant three-way interaction between Validity, Experiment, and Difficulty [*F*(1,27) = 9.8, *p* < 0.004]. This complex interaction was driven by a Validity effect (invalid > valid trials) only in the difficult discrimination conditions of Experiment 2 [IES mean: 62 ms; *t*(11) = 2.4, *p* = 0.036; see **Figure 1B**, right graph]. No other effect reached significance.

We further explored the Validity effect in Experiment 2 by evaluating costs vs. benefits for invalid/valid trials compared to the neutral cues in separate *t*-tests. We considered only the “hard” conditions of Experiment 2, averaging across ISIs. We tested for cueing costs in invalid trials (invalid > neutral) and for cueing benefits in valid trials (neutral > valid), using one tailed *t*-tests. These showed the expected costs of invalid trials [IES _invalid__-__neutral_: 122 ms, *t*(11) = 1.81; *p* < 0.049]; while the valid trials did not lead to any benefits and actually showed numerically lower performance than the neutral trials [IES _neutral__-__valid_: –60 ms, *t*(11) = –0.95; *p* > 0.8].

To summarize, the behavioral results demonstrated an effect of crossmodal semantic congruence on the processing of the subsequent visual targets and showed a role of the current task demands on this behavioral effect. Specifically, we found a significant difference between invalid vs. valid trials (IES_invalid_ > IES_valid_) only when the primary visual task had high demands (“hard” conditions of Experiment 2, see **Figure 1B**).

### fMRI Results

The main fMRI analysis compared “valid” and “invalid” trials with the aim of revealing any spatial effect of crossmodal semantic congruence on the processing of the subsequent visual targets; and assessed this under different task constraints: i.e., 0/250 ms ISI; easy/hard target discrimination; Experiment 1/2, with single vs. two Gabor patches in the target phase of the trial. The corresponding main effects and interactions were tested in a ANOVA that for each subject considered the contrast “invalid minus valid trials,” modeling the effects of ISI, Difficulty, and Experiment at the group level (see Materials and Methods, Magnetic Resonance Imaging).

**FIGURE 2:**
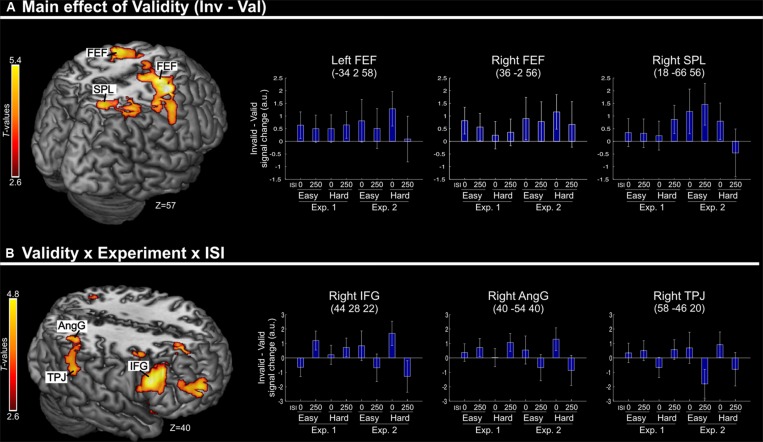
**(A)** Transversal section through a 3D rendering of the canonical MNI template showing activations for the main effect of Validity (invalid minus valid trials), revealing recruitment of the dorsal fronto-parietal attention network. The corresponding signal plots show greater activity for invalid vs. valid trials for all these regions, irrespective of the other experimental factors related to top–down task demands. **(B)** Transversal section through a 3D rendering of a canonical MNI template showing activations modulated by the interaction among Validity, Experiment, and ISI, revealing that crossmodal semantic congruence interacted with top–down task-related factors in the right ventral fronto-parietal attention network. For display purposes, all activation maps are displayed at a threshold of *p*-uncorrected < 0.005. Signal plots report “invalid minus valid” trials (error bars represent 90% C.I.) in arbitrary units (a.u.). SPL, superior parietal lobule; FEF, frontal eye-fields; IFG, inferior frontal gyrus; TPJ, temporo-parietal junction; AngG, angular gyrus.

Irrespective of task constraints, we found a main effect of “invalid > valid” trials in dorsal fronto-parietal regions, including the frontal eye-fields (FEF) bilaterally and the right superior parietal lobule (SPL, see **Figure 2A**; **Table 2**). As shown in the corresponding signal plots, these regions showed larger activity for invalid than valid trials across trial types (see positive effect sizes, on average, in these plots).

**Table 2:** Brain activations associated with the effect of Validity.

Contrast	Area	*P*-corrected	Cluster size	*T*-value	*x y z*
Main effect of Validity	R FEF	<0.001	3082	5.53	36 –2 56
	L FEF			4.39	-34 2 58
	R SPL	0.025	613	3.96	18 -60 56
Interaction: Validity ×ISI× Experiment	R IFG	0.003	899	4.80	44 28 22
	R TPJ	0.026	608	3.69	58 -46 20
	R AngG			3.49	40 -54 40

*MNI coordinates of the peak (*x, y, z*), cluster size (number of supra-threshold voxels, estimated at *p*-uncorrected = 0.005), *T*-values, and *p*-FWE-corrected values are shown for areas showing a significant main effect of Validity (invalid minus valid trials, see **Figure 2A**) and for the interaction among Validity, ISI, and Experiment (see **Figure 2B**). R/L, left/right hemisphere; FEF, frontal eye-fields; SPL, superior parietal lobe; IFG, inferior frontal gyrus; TPJ, temporo-parietal junction; AngG, angular gyrus.*

In contrast, in the ventral fronto-parietal cortex we found that the Validity effect was significantly modulated by the current task demands. Specifically, we found a 3-way interaction among Validity, Experiment, and ISI in the right inferior frontal gyrus (IFG) and in the right inferior parietal cortex, with a cluster comprising the temporo-parietal junction and the angular gyrus (TPJ and AngG, see **Figure 2B**; **Table 2**). The signal plots in **Figure 2B** show primarily a Validity effect (invalid > valid) for “ISI 0” trials of Experiment 2 (Bars 5 and 7, in these plots). In Experiment 1 the same Validity effect was larger for “ISI 250” than “ISI 0”. The finding of opposite effects in the two experiments may reflect that, in voxel-wise analyses, voxels with opposite effects of one factor under the two levels of the other factor will obtain high interaction-statistics and appear as peaks in the corresponding whole-brain map [e.g., the interaction “(A1 – A2) – (B1 – B2)” will be largest in voxels where: “A1 > A2” and “B2 > B1”].

Aside these main results concerning the effect of Validity and the interaction of this with the other experimental factors, we also tested for the effects of target discrimination Difficulty and cue-to-target ISI, irrespective of the in/-validity of the multisensory cues. For this we used two separate ANOVAs: one pooling Validity and ISI (testing for Difficulty and Difficulty-by-Experiment interactions) and the other pooling Validity and Difficulty (testing for ISI and ISI-by-Experiment interactions; see also Materials and Methods, Magnetic Resonance Imaging).

**FIGURE 3:**
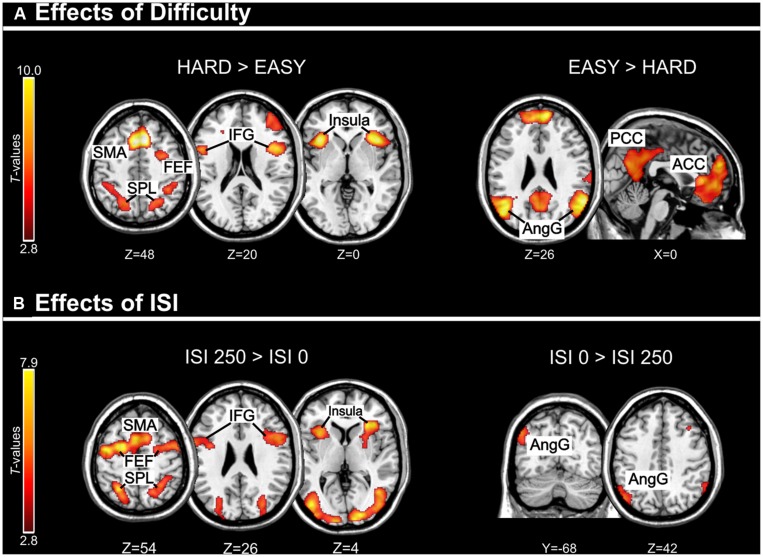
**(A)** Effects of Difficulty. Left panel: Axial sections showing the effect of Hard minus Easy trials, which recruited the dorsal fronto-parietal attention network, plus the insula and the IFG, bilaterally. Right panel: Axial and sagittal sections showing Easy trials, compared to Hard trials, recruited the default mode network. **(B)** Effects of ISI. Left panel: Axial sections showing the effect of long ISI250 minus short ISI0 trials, which recruited the dorsal fronto-parietal attention network, plus the insula and the IFG, bilaterally. Right panel: Coronal and axial sections showing the effect of ISI0 minus ISI250, which recruited the left angular gyrus. For display purposes, all activation maps were displayed at a threshold of *p*-uncorrected < 0.005. SPL, superior parietal lobule; FEF, frontal eye-fields; SMA, supplementary motor cortex; IFG, inferior frontal gyrus; PCC/ACC, posterior/anterior cingulate cortex; AngG, angular gyrus.

Across experiments, we found larger activation in dorsal fronto-parietal areas in “hard” compared to “easy” trials (see **Figure 3A**; panels on the left, including SPL and FEF), as well as for “long” compared to “short” ISIs (see **Figure 3B**, panel on the left; see also **Tables 3** and **4**). These comparisons also activated medial areas of the pre-motor cortex (SMA), as well as, the insula and the IFG, bilaterally (see **Figures 3A,B**, right panel). The medial pre-motor cortex often co-activates with other dorsal fronto-parietal areas and possibly plays a role in top–down attention control (e.g., Kastner and Ungerleider, 2001).

**Table 3:** Brain activations associated with the main effects of Difficulty.

Area	*P*-corrected	Cluster size	*T*-value	*x y z*
**Hard > Easy**		
SMA	<0.001	2792	9.97	8 18 52
R FEF	0.034	558	4.99	34 2 54
*L FEF*	*0.224*	*328*	*5.32*	*-24 2 54*
R SPL	0.007	766	4.25	24 -62 46
R aIPS	0.048	514	4.59	34 -32 36
L SPL	0.001	1066	4.63	-18 -64 46
L aIPS			4.38	-36 -38 36
R MFG	0.024	606	4.75	44 40 28
L Ins	0.002	935	7.45	-36 20 2
R Ins	<0.001	2209	7.10	42 20 -2
R IFG			5.91	48 6 18
*L IFG*	*0.079*	*454*	*3.48*	*-34 6 28*
**Easy > Hard**		
R AngG	<0.001	2002	7.60	60 -52 24
L AngG	<0.001	2511	7.19	-44 -54 20
PCC	<0.001	4410	5.61	-12 -40 40
ACC	<0.001	7251	7.02	8 56 24
L PHc	<0.001	1680	5.64	-24 -24 -22
Cereb	< 0.001	2821	5.99	28 -32 -36

*MNI coordinates of the peak (*x, y, z*), cluster size (estimated at *p*-uncorrected = 0.005), *T*-values, and *p*-FWE-corrected values for the areas activated by the comparison between “Hard vs. Easy”. Peaks *in italics* did not survive correction for multiple comparisons but are reported here if contralateral region survived correction. L/R, left/right hemisphere; SMA, supplementary motor area; FEF, frontal eye fields; SPL, superior parietal lobe; aIPS, anterior intra-parietal sulcus; MFG, middle frontal gyrus; Ins, insula; IFG, inferior frontal gyrus; AngG, angular gyrus; PCC, posterior cingulate cortex; ACC, anterior cingulate cortex; PHc, parahippocampal cortex; Cereb, cerebellum.*

**Table 4:** Brain activations associated with the main effects of ISI.

Area	*P*-corrected	Cluster size	*T*-value	*x y z*
**ISI 250 > ISI 0**		
SMA	<0.001	5426	6.16	-6 10 50
L FEF			6.85	-24 -2 56
R FEF			5.05	28 -4 50
L IFG			6.03	-56 4 40
R IFG			4.80	58 10 34
L OCC	<0.001	4569	7.86	-24 -96 12
L SPL			5.80	-26 -58 54
L aIPS			4.05	-44 -28 42
R OCC	<0.001	4097	6.61	22 -92 4
R SPL			4.82	24 -58 58
R aIPS			3.98	38 -28 40
R Ins	0.010	777	6.36	32 28 6
*L Ins*	*0.081*	*486*	*5.41*	-*32 18 4*
**ISI 0 > ISI 250**		
L AngG	0.041	577	4.63	-50 -68 42

*MNI coordinates of the peak (*x, y, z*), cluster size (estimated at *p*-uncorrected = 0.005), *T*-values, and *p*-FWE-corrected values for areas activated by the comparison between the two ISI conditions (0 vs 250 ms). Peaks *in italics* did not survive correction for multiple comparisons but are reported here if contralateral region survived correction. L/R, left/right hemisphere; SMA, supplementary motor area; FEF, frontal eye fields; IFG, inferior frontal gyrus; OCC, visual occipital cortex; SPL, superior parietal lobe; aIPS, anterior intra-parietal sulcus; Ins, insula; AngG, angular gyrus.*

The IFG cluster found for “hard > easy” overlapped considerably with the IFG cluster for “ISI 250 > ISI 0”, but this region of overlap was located more posteriorly than the IFG cluster involved in the significant 3-way interaction among Validity, Experiment, and ISI (see above, cf. **Figure 2A**). The opposite contrasts (“easy > hard” and “ISI 0 > ISI 250”) also revealed a common region in the angular gyrus bilaterally (see **Figures 3A,B**, right panels, and **Tables 3 and 4**). Additionally, the contrast comparing “easy > hard” trials showed significant effects in medial frontal and medial parietal areas, traditionally associated with the default-mode network (Raichle et al., 2001; Greicius et al., 2003; see **Table 3**), plus the cerebellum that is seldom reported in studies of attention but possibly plays a role in visuo-spatial orienting (see Striemer et al., 2015).

Finally, for Experiment 2 we compared the two trial types that included a picture congruent with the centrally presented sound (i.e., valid and invalid trials) vs. the neutral trials, when the sound was unrelated to either of the two pictures. The corresponding ANOVA considered the average of “valid and invalid” trails minus the neutral condition and modeled the effects of ISI and Difficulty at the group level (see also Materials and Methods, Magnetic Resonance Imaging). Irrespective of ISI and discrimination Difficulty, this revealed activity in Heschl’s gyrus, corresponding to the primary auditory cortex (right hemisphere: *x y z* = 60 -8 -2; cluster size = 935; *t* = 7.17; *p*-corrected = 0.001; left hemisphere: *x y z* = -54 -18 0; cluster size = 1214; *t* = 7.01; *p*-corrected < 0.001). An additional activation was found in the right superior occipital gyrus (*x y z* = 28 -78 42; cluster size = 1073; *t* = 4.05; *p*-corrected < 0.001), where the effect of the valid/invalid trials vs. neutral trials was larger for the hard than the easy trials. Since the acoustic characteristics of the sound in the valid and invalid trials were not matched with the neutral condition (i.e., cat/dog’s “meow/woof” vs. frog’s “croak”), here we will only underline the lack of activation in dorsal and ventral fronto-parietal regions without further discussing these effects in the auditory and occipital cortices.

## Discussion

The aim of the present study was to assess whether crossmodal interactions between semantically related, but spatially separated, audio–visual signals can affect the processing of subsequent visual targets. Specifically, we investigated these influences when the audio–visual stimuli were task-irrelevant and did not produce any low-level spatial cue for visuo-spatial orienting (e.g., physical changes at the same/opposite location of the visual target). Behavioral and imaging data showed that audio–visual semantic congruence can influence the processing of visual targets, modulating the activity in dorsal and ventral regions of the parietal and the premotor cortices. The localization of these effects most likely correspond to the dorsal (SPL and FEF, cf. **Figure 2A**) and ventral (rTPJ and rIFG, cf. **Figure 2B**) attention control networks (e.g., see Corbetta and Shulman, 2002).

At the behavioral level, we found that the subjects’ performance decreased when the visual targets were presented away from the location of the picture congruent with the sound, compared with targets presented at the same location (“invalid vs. valid” trials). A possible account of this crossmodal effect might be that the semantic relationship between the centrally presented sound and the congruent picture lead to a shift of visuo-spatial attention toward that picture. The presentation of the target on the opposite side would then require the re-orienting of visual attention from the location of the congruent picture to the position of the visual target, with corresponding behavioral costs (e.g., cueing costs for “invalid trials” in standard spatial cueing paradigms, see Posner and Cohen, 1984). However, the results of our study suggest that a more complex sequence of processes is taking place here. First, the behavioral data of Experiment 2 indicated that there was no cueing benefit for the congruent/valid conditions, but rather, that both invalid and valid trials lead to a reduction in performance compared to the “neutral” trials (central sound unrelated to either pictures). The lack of cueing-benefits appears at odds with previous studies that have demonstrated faster orienting toward the location of a target-picture congruent with a central sound and no costs when the sound was congruent with a distractor-picture (Iordanescu et al., 2008, 2010).

However, in these previous studies, the pictures were task-relevant, whereas in our study the pictures were fully task-irrelevant. Therefore, in previous studies any effect associated with crossmodal semantic congruence (e.g., enhanced representation of the congruent picture, Iordanescu et al., 2008) would match the current task set/target template: that is, stimulus-related semantic congruence and task-related, goal-driven attention work together to boost the processing of the same picture (i.e., the search target; see also Iordanescu et al., 2010). In contrast, in our study the objects displayed in the pictures were irrelevant; the subject’s only task was to judge the orientation of the Gabor patches presented after the audio–visual objects. We suggest that in this situation, the brain detected the semantic correspondence between the central sound and one of the two the pictures, registering the position of the congruent picture. However, because the task did not involve any object discrimination or spatial orienting toward that picture (cf. Iordanescu et al., 2008, 2010), goal-related attention control generated inhibitory rather than boosting signals, thus with opposite effects of semantic congruence and goal-directed attention. In this view, even on “valid” trials (congruent-picture on the same side as the Gabor-target), the pictures in our study would be more comparable with the distractor pictures than the target pictures used in previous search tasks that also combined pictures with semantically in-/congruent central sounds. Thus, the interplay between semantic congruency and task goal might explain the overall decrease of performance when the trial included a congruent picture (valid and invalid conditions) compared with trials containing a sound unrelated to both pictures (neutral condition). In the latter case, there would be no need to ignore and suppress the crossmodally enhanced object representation, making the discrimination of the subsequent visual targets faster.

Despite the lack of relationship between the object/picture associated with semantic congruence and the current task-goal may have reduced the processing the irrelevant pictures, our data showed that the presentation of the visual target in the hemifield opposite to the congruent-picture lead to several behavioral and imaging effects. Behaviourally, we found a further reduction of performance for invalid vs. valid trials but only in the “most difficult” conditions of Experiment 2 (finer orientation discrimination and additional distractor Gabor). Similarly, we found an effect of validity and task demand in our imaging results: activity in the ventral fronto-parietal network was modulated by the interaction between validity (invalid > valid) and task demands (see **Figure 2B**) while in the dorsal fronto-parietal network, the effect of validity was observed across all conditions (**Figure 2A**).

The imaging findings in the dorsal system indicate that, in spite of any object-related suppressive mechanism as discussed, the brain did register the location for the task-irrelevant picture coupled with the semantically congruent sound. Several recent studies have reported activation of dorsal fronto-parietal regions in response to salient visual stimuli, even when these were task-irrelevant (e.g., Bogler et al., 2011; Nardo et al., 2011; see also Schall and Hanes, 1993; Constantinidis and Steinmetz, 2001). In the context of multisensory spatial processing, Nardo et al. (2014) showed that the saliency of sounds in complex and naturalistic audio–visual video clips modulated activity in the posterior parietal cortex. This saliency-related modulation of activity in the parietal cortex was found only when the auditory stimuli were spatially congruent (i.e., on the same side) as the main visual event in the scene. Analogous with that study, we propose that here the semantic congruence between one picture and the centrally presented sound generated a processing priority bias in dorsal parietal regions (see Gottlieb et al., 1998; and Awh et al., 2012; Ptak, 2012, for reviews), which required updating when the task-relevant visual target was presented on the opposite side to congruent audio–visual pairs (invalid trials). While inhibitory interactions between object-related crossmodal processing and goal-related attention did not result in any behavioral benefit on valid trials, the spatial updating operations affected activity in the dorsal attention network and were observed using fMRI in all invalid conditions.

In contrast, the activation of the right ventral attention system (rIFG and rTPJ, see **Figure 2B**) was observed only when the visual discrimination task required top–down control to identify the task-relevant Gabor patch in Experiment 2. Many previous imaging studies have reported activation of the ventral attention network comparing invalid versus valid trials following predictive central cues (e.g., Arrington et al., 2000; Corbetta et al., 2000). While these effects might be associated with stimulus-driven shifts of spatial attention triggered by the onset of a stimulus at an unattended location, recent evidence indicates that the activation of the ventral system reflects a more complex interplay between the stimulus-driven signals and other factors associated with current task demands (e.g., Kincade et al., 2005; Indovina and Macaluso, 2007; Natale et al., 2009; see Corbetta et al., 2008, for a review). In the current study, the presence of a bilateral stimulation in the target phase of Experiment 2 lead to high demands on top–down control (see also overall low performance in Experiment 2 vs. Experiment 1, **Figure 1B**). Here, we suggest that the need of selecting one of the two Gabor patches based on *a priori* internal information (i.e., task instructions) produced top–down demands that interacted with any spatial priority bias associated with the congruent picture, leading to the activation of the right ventral network. This process might not strictly involve any shift of spatial attention but possibly entails the update of spatial predictions generated during the processing of the multisensory stimuli (Geng and Vossel, 2013; Macaluso and Doricchi, 2013; see also Shams and Beierholm, 2010, for a related and more formal framework).

It should be noted that such an expectation/prediction framework has been previously put forward in the context of endogenous spatial cueing, while here we suggest that the initial priority bias was generated by the irrelevant and non-predictive (audio–visual) stimuli. This difference could explain the finding here that ventral network activity was observed only at the short ISI (cf. interaction between Validity, Experiment, and ISI; **Figure 2B**). Unlike endogenous cues that are typically associated with long-lasting spatial effects, here we would expect any bias generated by the irrelevant audio–visual stimuli to be relatively short-lived, and therefore, any process triggered by the interaction between these crossmodal effects and top–down control signals (i.e., identification and selection of the target Gabor) would take place only when the visual targets were presented in close temporal proximity of the audio–visual stimuli, i.e., at short ISIs. It should be noted that at the short ISI, we also observed an overall reduction of behavioral performance (cf. main effect of ISI). Because the visual targets were presented at the same location as the irrelevant pictures, this suggests a possible role of forward masking when ISI = 0. However, the behavioral data also showed orientation discrimination accuracies up to 95% (see **Table 1**, “easy” conditions) demonstrating the targets were well visible, incompatible with a major role of forward masking.

Aside from the specific mechanisms and interpretations that we proposed above, the current findings provide us with novel insights about the interplay between top–down and bottom–up signals in the context of multisensory processing. Extensive research has highlighted the complex interplay between these types of signals in multisensory integration (e.g., see Talsma et al., 2010, for a review). In the current study, we did not measure the crossmodal binding between the sound and the congruent picture nor of perceived sound location shift toward the congruent picture. However, we observed changes in visuo-spatial processing (valid vs. invalid trials) that provide us with an indirect measure that semantic crossmodal interactions affect how attention is allocated in the visual space. Most importantly, our experimental setup allowed us to demonstrate these effects in the absence of any physical low-level change or task-related association between the crossmodal cues the subsequent visual target. In this setup, the results cannot be attributed to any direct effect of purely stimulus-driven or purely goal-driven attention. In contrast, previous studies typically relied on physical changes of the sensory input at peripheral locations (e.g., spatial cueing paradigms, Santangelo et al., 2008a,b; see Spence and Santangelo, 2009; and Spence, 2010 for a review; see also Van der Burg et al., 2008; Matusz and Eimer, 2011); and/or on the existence of some goal-related relationship between the audio–visual stimuli and the to-be-judged visual stimuli (e.g., Matusz et al., 2015). In the former case, stimulus-driven signals are likely to play a direct role, with the spatial and/or temporal correspondence of the sensory changes acting as the main cue triggering associations across modalities (see Santangelo et al., 2008a,b; Van der Burg et al., 2008). Here, the presentation of two pictures and a centrally presented sound in all conditions eliminates any such low-level cues and ensures that crossmodal influences on visual attention could be specifically attributed to the semantic correspondence between the auditory and visual input.

Uncoupling the audio–visual stimuli (animal pictures and sounds) from the sole visual task (Gabor orientation discrimination) also enabled us to demonstrate crossmodal semantic influences in the absence of any goal-related signal linking the audio–visual stimuli with the task-relevant visual target. Such task-based relationships characterized previous studies where the crossmodally enhanced visual object was also the target of the search task (Iordanescu et al., 2008, 2010) or shared some task-relevant feature with the search target (Matusz et al., 2015). We extend these previous results by demonstrating that crossmodal semantic congruence affects visual attention despite opposing top–down task constraints. These results are in line with results from previous studies indicating that crossmodal effects can occur in the absence of goal-directed attention toward the audio–visual stimuli (Alsius et al., 2005; see also Santangelo and Spence, 2007; but note that in these studies physical changes, rather than semantic congruence, might have contributed to associate the auditory and visual signals; see also Van der Burg et al., 2012). Nonetheless, other studies have found that increasing the demands of a primary task, while presenting participants with multisensory stimuli, can reduce the interaction between the multisensory input (e.g., Alsius et al., 2005; see also Talsma et al., 2010, for a review). Contrary to these studies, we found several crossmodal effects only when the demands of the primary visual task were high (cf. behavioral results and the pattern of activation in the ventral attention system in Experiment 2). A possible reason for these differences is that here task demands specifically concerned the target phase of the trial and not the resources available to process the audio–visual stimuli. Thus, task demands can have multifaceted consequences on the processing of multisensory stimuli, including suppression (Alsius et al., 2005; using a dual-task approach), no effect (Matusz et al., 2015; presence of competing distractors in a crossmodal interference paradigm), and selective influences only under high demands (the current study, where crossmodal signals interact with attention control operations under high demands only, as discussed above); see also Koelewijn et al. (2010) and Talsma et al. (2010) for reviews on the impact of task demands on multisensory processing.

## Conclusion

The present study demonstrated that crossmodal semantic congruence between spatially separated audio–visual stimuli can affect visual-spatial attention control. We found these crossmodal effects in the absence of any physical change that might capture visual attention in a direct bottom–up manner, and of any goal-related relationship between the audio–visual stimuli and the visual targets; that is, with fully task-irrelevant audio–visual stimuli. We discussed these effects in relation to multiple signals associated with the processing of irrelevant audio–visual stimuli and the top–down task demands of the primary visual task. We propose that the semantic congruence between the task-irrelevant audio–visual stimuli generates processing biases that require updating when a subsequent task-relevant visual target is presented at a different location. We relate these updating operations to saliency representations in the dorsal attention network and with the interplay between stimulus- and task-related signals in the ventral attention network. We conclude that crossmodal semantic congruence can affect visual-spatial processing in the absence of any direct bottom–up or goal-related influences, and highlight the role of the fronto-parietal attention control networks in mediating the effect of multisensory processing on visual attention.

## Conflict of Interest Statement

The authors declare that the research was conducted in the absence of any commercial or financial relationships that could be construed as a potential conflict of interest.
